# The influence of workplace stressors on negative attitudes toward long-term male parental leave: a cross-sectional study in Japan

**DOI:** 10.3389/fpubh.2025.1507607

**Published:** 2025-06-25

**Authors:** Hiromi Ono

**Affiliations:** Faculty of Human Sciences, University of Tsukuba, Tsukuba, Japan

**Keywords:** male parental leave, sense of unfairness, workplace stressors, anger, negative feelings, work-life balance

## Abstract

**Objective:**

Although work–life balance measures have become an important theme in corporate personnel resource management, they have not been thoroughly examined from the perspective of perceived fairness within Japanese organizations. This study developed a model hypothesizing that supervisors’ awareness of interpersonal justice is negatively associated with workplace stressors, while workplace stressors are positively associated with anger through situations that evoke a sense of unfairness related to male parental leave.

**Methods:**

A web panel survey was conducted through an Internet research company. Four hundred valid responses were obtained (200 men and 200 women; M_age_ = 40.25 ± 10.59 years). Participants completed measures of anger, sense of unfairness, workplace stressors, and supervisors’ interpersonal justice.

**Results:**

Men were more likely than women to harbor feelings of anger regarding their male coworkers’ uptake of long-term parental leave. Women were more likely than men to harbor resentment toward male coworkers who take parental leave without fully dedicating themselves to childcare. The goodness-of-fit analysis indicated a good model fit.

**Conclusion:**

Workplace stressors are positively associated with anger through a sense of unfairness regarding male parental leave. In Japan, it is easy to think that child-rearing is the responsibility of women, and it is likely that colleagues in the workplace will have negative feelings toward men who take long-term parental leave. In addition, since the interpersonal fairness of supervisors is negatively associated with workplace stressors, it can be concluded that supervisors play an important role in workplaces where men take long periods of parental leave. In the future, if the number of men taking long-term parental leave increases, studies focusing on employees whose male coworkers have taken long-term parental leave will be possible.

## 1 Introduction

### 1.1 Current status of male parental leave in Japan

The Revised Act on Childcare Leave/Caregiver Leave, which encourages men to take parental leave, was enacted in June 2021 in Japan. Promoting male parental leave has become a focus in corporate work–life balance (WLB) (1). According to a report released by UNICEF in 2021, Japan ranked first in terms of the adequacy of its parental leave and childcare policies among 41 countries in the OECD and EU (2). This is because Japan offers the longest period of parental leave for fathers, which is generally until the child reaches the age of one, and wages are guaranteed through parental leave benefits. However, the current situation in Japan is that the system is not being fully utilized. While over 80% of women who work at Japanese corporations take parental leave, the rate for men, despite being at a record high, is only about 30% (3). Gender differences are also evident in the duration of parental leave: over 90% of women take leave lasting 6 months or more, while approximately 40% of men take less than 2 weeks (3). Despite the Japanese Government’s apparent desire to encourage fathers to take parental leave, this system has diverged significantly from the Nordic and German systems to which it was originally compared (4). The patchwork situation in which Papa Mama Childcare Leave Plus, a system modeled on the Papa Quota, did not work as intended was seen as a systemic problem, although the situation was left untouched allowing mothers alone to extend their leave until the child turns two-years-old (4). This situation can be attributed to Japan’s work culture and gender roles. Japanese men have longer paid work hours than men in other countries (5). In 2021, among the 25–54 age group, there were 4.9 times more women employed in part-time positions than men. Japan’s gender wage gap of 22 percent was the highest among G7 countries, and men worked 29 percent more hours than women in Japan (6). In addition, in Japan, women with children six-years-old or younger spend close to four times longer on housework than their husbands (7).

In this study, parental leave for men includes both the leave stipulated by law and also paid parental leave at the time of birth, which is provided independently by some companies. Ono (8) defines long-term parental leave for men as 1 month or longer because it is necessary to adjust work before taking parental leave and because it is believed that men will be dedicated to childcare daily during this leave. Long-term parental leave for men in this study also follows Ono’s (8) definition.

### 1.2 Studies on male parental leave

Male parental leave has been addressed within the context of WLB research. Corporations whose male employees use the parental leave program also implement multiple WLB support measures (9). In workplaces where male employees use the parental leave system, corporate management treats their employees appropriately (10). A workplace environment that makes it easy to take parental leave is cited as a factor influencing men to take parental leave (11–13). Thus, studies on encouraging men to take parental leave have focused on systems and workplace environmental aspects.

Family-related leave tends to lead to lower performance evaluations (14) and is often perceived as indicating low organizational commitment (15). Fearing such negative evaluations, men tend to be reluctant to take parental leave (16, 17). One of the reasons why men in Japan are reluctant to take parental leave is thought to be the existence of widespread ignorance, whereby people assume that others are more negative about male parental leave than they themselves are (18). However, the findings also show that men who took parental leave reported increased personal growth, job performance, and workplace satisfaction (19). Taking leave also leads to efficient business performance upon return (20–22) and changes in attitudes concerning work and home responsibilities (20, 21), such as increased involvement in childcare (23). More attention is thus being paid to the positive aspects of men taking parental leave.

However, “Increased burden placed on others” and “causing trouble at the workplace” were also cited as negative impressions held toward men who take long-term parental leave (24, 25). When a male employee takes long-term parental leave, his supervisor tends to harbor negative feelings if they feel burdened by the excessive workload (26). There is also evidence that men who take long-term parental leave become anxious owing to concerns about others’ perspectives and guilt over placing a burden on their colleagues (20). Further, a sense of unfairness can arise within companies promoting male parental leave (8). Three factors that evoke such emotions toward a male coworker who takes long-term parental leave are “lack of involvement in childcare,” “receiving generous benefits,” and “increase in one’s workload” (27). Hosomi and Sekiguchi (28) surveyed government and other public employees and found that employees who work long overtime hours have higher workload expectations (the degree to which an individual predicts that their own workload will increase as a result of a coworker using the WLB programs) than employees who do not work long hours, as well as decreased support for WLB programs.

According to Adams’ (29) equity theory, equity is formed if the ratio between remuneration/benefits that one has received (“outputs”) and their contribution (“inputs”) matches the ratio of inputs and outputs of others. Anger and other negative emotions are evoked if there is inequity, where one is subject to inequitable treatment and thus suffers a disadvantage. Although wages and treatment primarily correspond to remuneration, seeing others enjoy the benefits of employee welfare and WLB programs may also become a basis for judging if a comparison between oneself and others is fair or not. Although WLB measures have become an important theme in corporate personnel resource management, they have not been thoroughly examined from the perspective of perceived fairness within an organization.

### 1.3 Relation to stress

The findings of an interview survey conducted by Ono (30) that focused on coworkers’ psychology revealed that negative feelings toward a male employee taking long-term leave arise from a sense of unfairness and increased workload but are mitigated by supervisor support. According to Cooper and Marshall’s (31) Occupational Stress Model, workplace stressors cause depression, job dissatisfaction, and decreased motivation. Therefore, workplace stressors can make people more sensitive to the interests of others and their own disadvantages, which can lead to a sense of unfairness.

Organizational justice is defined as a subsidiary perception of fairness within an organization that includes two components: distributive justice, which concerns the fairness of resource distribution outcomes, and procedural justice, which relates to the fairness of the processes by which those outcomes are determined (32). Interactional justice, which pertains to interpersonal treatment during these processes, is also considered a part of organizational justice (33). Colquitt (34) further divided interactional justice into two dimensions: interpersonal justice, which refers to how supervisors treat subordinates, and informational justice, which pertains to information disclosure. Low organizational justice can increase the risk of health problems in the workplace (32) and is associated with minor mental disorders (35). Associations between organizational justice and cardiovascular disease have been reported, indicating that organizational justice is directly related to absenteeism due to illness and indirectly related to turnover rates (36). Interactional justice, which includes the perception of respectful and dignified treatment from supervisors, can reduce loneliness and enhance psychological safety in remote and hybrid work contexts (37). Procedural and distributive justice also contribute to this dynamic by promoting a sense of control and fairness in resource distribution and decision-making processes, which may decrease perceived exclusion (38). Thus, interpersonal justice is positively associated with workplace stressors.

### 1.4 Study purpose and hypotheses

Based on the preceding discussion, this study investigates the interrelated associations among supervisors’ interpersonal justice, workplace stressors, and emotional responses related to male parental leave. Specifically, it proposes a model in which supervisors’ interpersonal justice is negatively associated with workplace stressors, while workplace stressors are positively associated with anger through situations that evoke perceptions of unfairness. This study examines three hypotheses.

1. Supervisors’ interpersonal justice is negatively associated with workplace stressors.
2. Workplace stressors are positively associated with situations that evoke a sense of unfairness regarding male parental leave.
3. Situations that evoke a sense of unfairness regarding male parental leave are positively associated with feelings of anger.

## 2 Materials and methods

### 2.1 Survey participants and procedures

A web panel survey was conducted through an Internet research company. A questionnaire was distributed to monitor members registered with the company, and those who clicked the “I agree” button on the survey start screen responded. Four hundred valid responses (200 men and 200 women, mean age = 40.25 ± 10.59 years) were obtained from full-time employees aged 20 to 60 years, including those working in private companies and individuals in non-corporate management positions or managerial roles at the section manager level. The Internet research company conducted data cleaning to exclude inauthentic responders and those who do not spend enough time answering the questions. The survey was conducted from September 10–12, 2024.

### 2.2 Survey items

The survey items are described below. The survey was titled, “A questionnaire regarding the workplace and work.”

#### 2.2.1 Anger

Four “anger” items from Oda et al.’s (39) checklist of emotions and arousal were used. The instructions were as follows: Imagine that you have a male coworker (neither a supervisor nor a subordinate) at your workplace who will take or has taken parental leave for more than a month. How applicable are the statements below to your feelings at that time? Choose the option that you feel best applies. Responses were given on a four-point scale, ranging from 1 (*not applicable at all*) to 4 (*highly applicable*).

#### 2.2.2 Situations that evoke a sense of unfairness regarding male parental leave

Ono’s (27) scale of situations that evoke a sense of unfairness regarding male parental leave was used (seven items for “lack of involvement in childcare,” eight items for “receiving generous benefits,” and seven items for “increase in one’s workload”). The instructions were as follows: “Imagine that you have a male coworker (not a supervisor or subordinate) at your workplace, who will take or has taken, parental leave for more than a month. To what extent do you perceive a sense of unfairness in the various situations described below? Choose the option that you feel best applies. Responses were given on a five-point scale, ranging from 1 (*not at all*) to 5 (*very much*).

#### 2.2.3 Workplace stressors

The revised workplace stress scale by Kosugi et al. (40) was used, including 12 items for “stressors due to qualitative load” and eight items for “stressors due to quantitative load.” The instructions were as follows: “Read the question items in order and choose one option that applies to you.” Responses were given on a five-point scale from 1 (*not applicable at all*) to 5 (*highly applicable*).

#### 2.2.4 Supervisors’ interpersonal justice

Four items for “interpersonal justice” from the Japanese version of the Organizational Justice Scale by Shibaoka et al. (41) were used. The instructions were as follows: “Regarding your supervisor (an individual with the authority to determine your personal evaluation), choose one option that applies.” Responses were given on a five-point scale from 1 (*hardly applicable at all*) to 5 (*definitely applies*).

#### 2.2.5 Demographics

Besides the questions noted above, respondents were asked to provide information about their title, industry, type of job, marital status, parental status, and any experience with taking parental leave.

### 2.3 Statistical analyses

Covariance structure analysis was performed using AMOS 28.0 (IBM Corp., Armonk, NY, United States) to test the hypothesized path model. The model consisted of four levels: supervisors’ interpersonal justice (Level 1), two workplace stressor factors (Level 2), three situational factors that evoke a sense of unfairness regarding male parental leave (Level 3), and anger (Level 4). In conducting the analyses, paths were specified from Level 1 to Levels 3 and 4, and from Level 2 to Level 4. Model fit was evaluated using standard fit indices and reference cut-off criteria commonly used in structural equation modeling.

## 3 Results

### 3.1 Respondent attributes

Table 1 shows participants’ demographics. There were 264 people (66.0%) without children, 16 people (4.0%) with the youngest child aged 0 to 1, 19 people (4.8%) with the youngest child aged 2 to 5, 34 people (8.5%) with the youngest child aged 6 to 12, and 67 people (16.8%) with the youngest child aged 13 or older. Regarding job titles, non-management positions accounted for 364 (91.0%), while management positions accounted for 36 (9.0%). The number of employees at the respondents’ companies was as follows: fewer than 100 employees: 143 (35.8%); 100 to 299 employees: 60 (15.0%); 300 to 499 employees: 33 (8.3%); 500 to 999 employees: 38 (9.5%); 1,000 to 2,999 employees: 38 (9.5%); 3,000 to 4,999 employees: 16 (4.0%); and 5,000 or more employees: 72 (18.0%). The industries of the companies where the respondents are employed are as follows: manufacturing (110 employees, 27.5%), IT and communications (48 employees, 12.0%), distribution and trading (35 employees, 8.8%), finance and insurance (23 employees, 5.8%), construction and real estate (45 employees, 11.3%), services (93 employees, 23.3%), and others (46 employees, 11.5%). The job types were sales (49, 12.3%), planning (7, 1.8%), administrative (142, 35.5%), specialized (31, 7.8%), sales and service (43, 10.8%), IT (26, 6.5%), technical (65, 16.3%), and other (37, 9.3%).

**Table 1:** Respondents’ demographics.

Age group	Childless men	Fathers	Childless women	Mothers
	*n*	*n*	*n*	*n*
20s	48 (2)	2 (1)	41 (0)	9 (0)
30s	33 (1)	17 (2)	36 (0)	14 (1)
40s	24 (3)	26 (8)	26 (1)	24 (0)
50s	22 (7)	28 (10)	34 (0)	16 (0)

The numbers in parentheses indicate those with positions in corporate management.

### 3.2 Examination of scale reliability and common method bias

A confirmatory factor analysis was performed for each subscale, and the mean variance extracted (AVE), construct reliability (CR), and Cronbach’s alpha coefficient (*α*) were calculated to assess validity and reliability (Table 2). To verify the influence of common method bias, Harman’s single-factor test was conducted. Exploratory factor analysis was performed on all observed variables, and, as expected, seven factors were extracted. Further, the proportion of variance in all observed variables explained by the first factor, which had the largest eigenvalue, was 26.5%. Based on the above test results, we conclude that the possibility of common method bias in this sample is low.

**Table 2:** Fitness of each scale, AVE, CR, and Cronbach’s *α*.

Variables	CFI	TLI	RMSEA	SRMR	AVE	CR	α
Anger	0.980	0.939	0.224	0.013	0.867	0.963	0.963
Lack of involvement in childcare	0.945	0.917	0.147	0.038	0.657	0.930	0.929
Receiving generous benefits	0.884	0.838	0.151	0.062	0.500	0.886	0.885
Increase in one’s workload	0.908	0.862	0.183	0.049	0.643	0.926	0.926
Stressors due to qualitative load	0.870	0.842	0.109	0.058	0.413	0.891	0.890
Stressors due to quantitative load	0.928	0.899	0.102	0.051	0.424	0.849	0.841
Supervisors’ interpersonal justice	0.998	0.994	0.054	0.008	0.740	0.919	0.917

### 3.3 Comparison of scale scores

Because the number of items corresponding to the subscales of each scale differed, the assessment values of each corresponding item were summed, divided by the number of items, and the resulting quotient was used as the subscale score. The mean and standard deviation were calculated for each of the four groups (childless men, fathers, childless women, and mothers) according to gender and parental status, and a two-way analysis of variance was performed (Table 3). The results showed a significant main effect of gender on anger [*F*(1, 396) = 7.64, *p* = 0.006, ηp^2^ = 0.02], with men scoring higher than women. Concerning situations that evoke a sense of unfairness regarding male parental leave, the main effect of gender was significant for “lack of involvement in childcare” [*F*(1, 396) = 17.22, *p* < 0.001, ηp^2^ = 0.04], with women scoring higher than men. The main effect of parental status was significant [*F*(1, 396) = 2.63, *p* = 0.041, ηp^2^ = 0.01], with parents scoring higher than childless men and women, regardless of gender. No interactions or main effects were found for “receiving generous benefits.” Significant interactions were found for “increase in one’s workload” [*F*(1,396) = 6.10, *p* = 0.014, ηp^2^ = 0.02]. A simple main effect test revealed that the effect of gender was significant for the parent group [*F*(1,396) = 4.34, *p* = 0.038, ηp^2^ = 0.01], with fathers scoring higher than mothers. Regarding workplace stressors, significant interactions were found only for “stressors due to quantitative load” [*F*(1,396) = 4.50, *p* = 0.035, ηp^2^ = 0.01]. A simple main effect test revealed that the effect of gender was significant for the parent group [*F*(1,396) = 10.60, *p* = 0.001, ηp^2^ = 0.03], with fathers scoring higher than mothers. No interactions or main effects were found in connection with supervisors’ interpersonal justice.

**Table 3:** Means, standard deviations, and analysis of variance results for each scale by group.

Variables	Childless men		Fathers		Childless women		Mothers		Main effect		Reciprocal interaction
	Mean	*SD*	Mean	*SD*	Mean	*SD*	Mean	*SD*	Gender	Child	Gender × child
Anger	1.48	0.68	1.51	0.76	1.42	0.59	1.20	0.50	7.64^**^	-	-
Lack of involvement in childcare	2.06	0.77	2.21	0.77	2.38	0.81	2.57	0.79	17.22^***^	4.21^*^	-
Receiving generous benefits	2.26	0.79	2.42	0.67	2.28	0.64	2.26	0.64	-	-	-
Increase in one’s workload	2.49	0.84	2.69	0.81	2.63	0.77	2.40	0.74	-	-	6.10^*^
Stressors due to qualitative load	2.77	0.75	2.72	0.75	2.84	0.71	2.68	0.68	-	-	-
Stressors due to quantitative load	2.71	0.78	2.86	0.84	2.63	0.72	2.44	0.71	-	-	4.50^*^
Supervisors’ interpersonal justice	3.47	0.87	3.39	0.90	3.35	0.98	3.58	0.88	-	-	-

^*^p < 0.05, ^**^p < 0.01, ^***^p < 0.001, - = non-significant.

### 3.4 Inter-factor correlations

Correlations were calculated between each factor (Table 4). Anger was significantly positively correlated with “lack of involvement in childcare,” “receiving generous benefits,” “increase in one’s workload,” “stressors due to qualitative load,” and “stressors due to quantitative load” and significantly negatively correlated with supervisors’ interpersonal justice. Regarding the relationship between situations that evoke a sense of unfairness regarding male parental leave and workplace stressors, “lack of involvement in childcare” and “receiving generous benefits” were significantly positively correlated with the two subscales of workplace stressors. “Increase in one’s workload” was significantly positively correlated with “stressors due to qualitative load” and “stressors due to quantitative load.” Regarding the relationship between workplace stressors and supervisors’ interpersonal justice, “stressors due to qualitative load” was significantly negatively correlated with supervisors’ interpersonal justice. A significant negative correlation was found between “stressors due to quantitative load” and supervisors’ interpersonal justice.

**Table 4:** Correlation analysis results.

Variables	1	2	3	4	5	6	7
1 Anger	-	0.25^**^	0.24^**^	0.27^**^	0.23^**^	0.26^**^	−0.29^**^
2 Lack of involvement in childcare		-	0.37^**^	0.60^**^	0.24^**^	0.25^**^	−0.19^**^
3 Receiving generous benefits			-	0.58^**^	0.28^**^	0.25^**^	−0.12^*^
4 Increase in one’s workload				-	0.34^**^	0.41^**^	−0.20^**^
5 Stressors due to qualitative load					-	0.45^**^	−0.44^**^
6 Stressors due to quantitative load						-	−0.23^**^
7 Supervisors’ interpersonal justice							-

^*^p < 0.05, ^**^p < 0.01.

### 3.5 Evaluation of model fit by covariance structure analysis

A path analysis was performed through covariance structure analysis based on a model that hypothesized the following: supervisors’ interpersonal justice was negatively associated with workplace stressors, such as “stressors due to qualitative load” and “stressors due to quantitative load”; workplace stressors were positively associated with situations that evoked a sense of unfairness regarding male parental leave, such as “lack of involvement in childcare,” “receiving generous benefits,” and “increase in one’s workload”; and these situations, in turn, were positively associated with anger.

**Figure 1:**
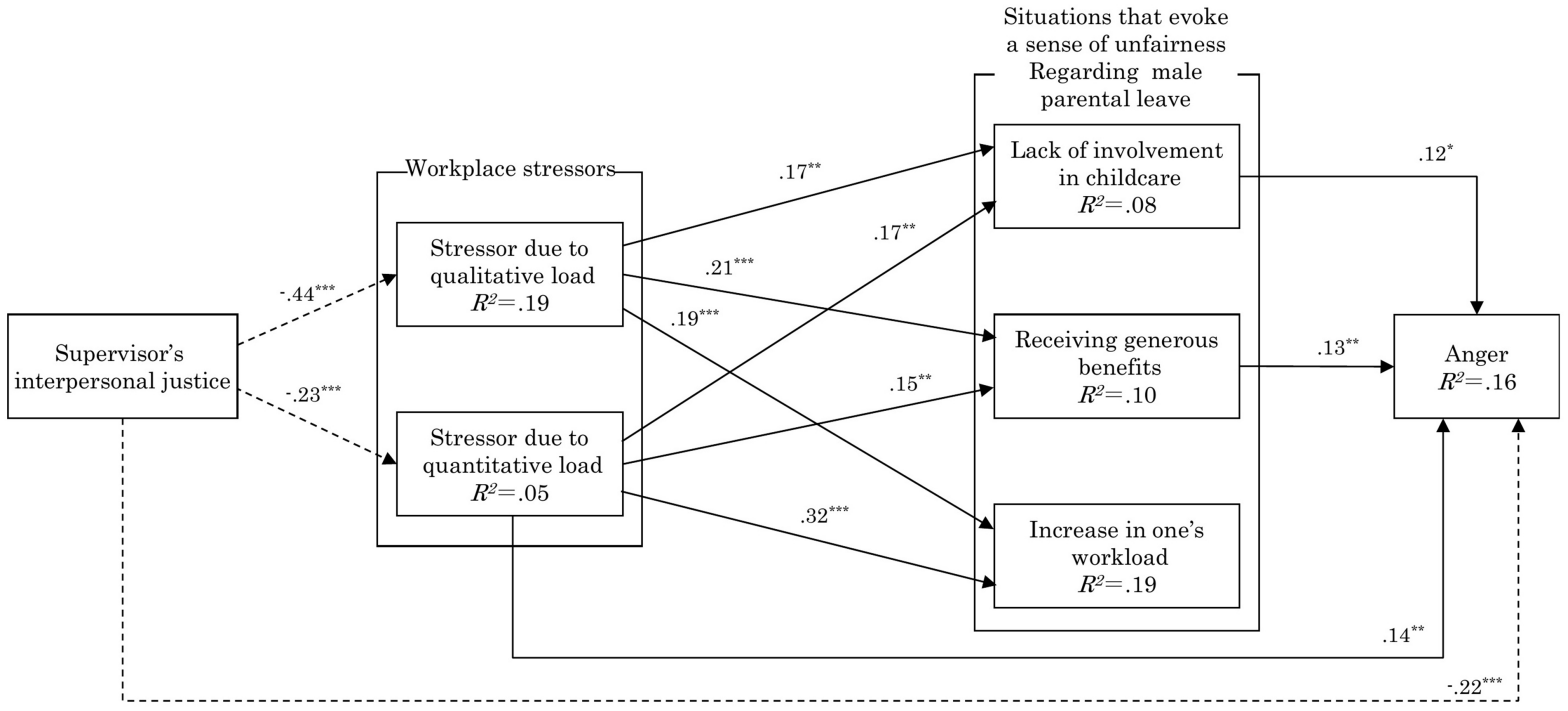
Path analysis results. ^***^p < 0.001, ^**^p < 0.01, ^*^p < 0.1. Solid lines indicate positive paths and dashed lines indicate negative paths.

Because the results showed some non-significant paths, I removed them and conducted a re-analysis. The final goodness-of-fit of the model was GFI = 0.996, AGFI = 0.979, CFI = 1.000, TLI = 0.999, RMSEA = 0.010, and SRMR = 0.019, indicating a good model fit. Figure 1 shows the standardized solution as a path coefficient. The analysis showed a significant negative path from supervisors’ interpersonal justice to “stressors due to qualitative load” (*β* = −0.44, *p* < 0.001) and “stressors due to quantitative load” (β = −0.23, *p* < 0.001), as well as a direct significant negative path to anger (*β* = −0.22, *p* < 0.001). From “stressors due to qualitative load,” a significant positive path was observed to “lack of involvement in childcare” (*β* = 0.17, *p* < 0.01), “receiving generous benefits” (*β* = 0.21, *p* < 0.001), and “increase in one’s workload” (β = 0.19, *p* < 0.001). From “stressors due to quantitative load,” a significant positive path was observed to “lack of involvement in childcare” (*β* = 0.17, *p* < 0.01), “receiving generous benefits” (β = 0.15, *p* < 0.01), and “increase in one’s workload” (β = 0.32, *p* < 0.001), as well as a direct significant positive path to anger (β = 0.14, *p* < 0.01). A significant positive path was found from “lack of involvement in childcare” (β = 0.12, *p* < 0.05) and “receiving generous benefits” (β = 0.13, *p* < 0.01) to anger, but no significant path was confirmed from “increase in one’s workload” to anger.

## 4 Discussion

### 4.1 Characteristics of scale scores by gender and parental status

Comparing the scale scores by gender and parental status revealed that men had significantly higher scores for anger than women, indicating that men were more likely to harbor feelings of anger regarding their male coworkers’ uptake of long-term parental leave. The rate of men taking parental leave in Japan exceeded 10% in 2020 and currently hovers around 30%. Since many men lack experience taking parental leave, it can be inferred that men are likely to harbor negative feelings toward the few men who take long-term parental leave, despite being of the same gender.

Concerning situations that evoke a sense of unfairness regarding male parental leave, the scores for “lack of involvement in childcare” were significantly higher for women than men. This suggests that when male coworkers take long-term parental leave, women tend to harbor greater feelings of resentment than their male counterparts in situations in which their coworkers are not particularly dedicated to childcare. In Japan, women with children six-years-old or younger spend close to four times longer on housework than their husbands (7), indicating that the burden of housework and childcare is still placed disproportionally on women. Moreover, in a studies by Ono that targeted working fathers (24) and mothers (25), factors such as “increased burden placed on others” and “causing trouble at the workplace” were commonly cited for both genders. In addition to these, working women held a negative image in the form of “vacation existing as a formality only” (25). Given these factors, women are more likely to harbor resentment than men toward male coworkers who take parental leave without fully dedicating themselves to childcare.

Scores for “lack of involvement in childcare” were higher in parents of both genders than in childless men and women. This indicates that when a male coworker takes long-term parental leave, in situations in which the coworker is not closely involved with childcare, his coworkers who have children of their own are more likely to perceive a greater sense of unfairness than childless coworkers. Many parents know from experience that childrearing is challenging, making them more likely to view a male employee’s lack of dedication during parental leave negatively. Fathers had significantly higher scores for “increase in one’s workload” than mothers. Few men opt to take parental leave even after a child is born; in this study, only nine men had taken such leave. Thus, when men see their workload increase due to a coworker taking parental leave, they likely perceive it as unfair.

Concerning workplace stressors for the parent group, the scores for fathers were higher than those for mothers, but only for “stressors due to quantitative load.” This indicates that among workers who have children, men have a greater volume of work than women, with an excessive burden placed on them. The “mommy track” phenomenon is evident in Japan, where female workers are pressured to leave their career paths after giving birth (42). Japanese corporations often assign secondary roles to women and limit their workloads.

### 4.2 Model examination

This study investigated a model hypothesizing that supervisors’ interpersonal justice was negatively associated with workplace stressors, such as “stressors due to qualitative load” and “stressors due to quantitative load”; that workplace stressors were positively associated with situations that evoked a sense of unfairness regarding male parental leave, such as “lack of involvement in childcare,” “receiving generous benefits,” and “increase in one’s workload”; and that situations that evoked a sense of unfairness regarding male parental leave were positively associated with anger. The results confirmed the model’s goodness-of-fit.

First, supervisors’ interpersonal justice was negatively associated with “stressors due to qualitative load” and “stressors due to quantitative load.” Therefore, Hypothesis 1 was supported. This finding aligns with Ono (30), who suggested that workplace stress factors are influenced by superiors’ responses. Organizational justice, which includes interpersonal justice, is related to health problems in the workplace (32) and mild mental illness (35). The perception of respectful and dignified treatment from supervisors can increase psychological safety. The current findings align with these earlier studies.

Second, both “stressors due to qualitative load” and “stressors due to quantitative load” were positively associated with all situational factors that evoke a sense of unfairness regarding male parental leave, such as “lack of involvement in childcare,” “receiving generous benefits,” and “increase in one’s workload.” Therefore, Hypothesis 2 was supported. This result coincides with Ono’s findings (26, 30), which indicate that having too much work leads to resentment and negative feelings toward men who take long-term parental leave. Workplace stressors can cause negative psychological effects (31), and this study showed that they also tend to evoke a sense of unfairness in men who take long-term parental leave. In addition, among the workplace stressors, “stressors due to quantitative load” was directly positively associated with anger. This aligns with the findings of Hosomi and Sekiguchi (28), who indicated that employees who work long overtime hours are more negative about their coworkers who use the WLB programs than employees who do not work long hours.

Third, of the situations that evoke a sense of unfairness regarding male parental leave, “lack of involvement in childcare” and “receiving generous benefits” were positively associated with anger, while “increase in one’s workload” was unrelated to anger. Therefore, Hypothesis 3 was partially supported. “Lack of involvement in childcare” pertains to the personal circumstances of men who have taken parental leave and does not directly relate to their coworkers. However, if men taking parental leave are perceived as simply taking a vacation without fulfilling its primary purpose, coworkers may view the situation as unreasonable and feel anger. “Receiving generous benefits” reflects an organization’s measures and shows that those taking parental leave gain benefits, which could foster anger among coworkers. However, an “increase in one’s workload” reflects the individual’s situation rather than that of the male coworker taking parental leave. Ono (30) indicated that if an individual is burdened with too much work, they are likely to harbor negative feelings, such as resentment, toward male coworkers who take extended parental leave. The current findings suggest that “stressors due to quantitative load” not only causes anger through “increase in one’s workload” but may also be directly positively associated with anger.

This study showed that workplace stressors are positively associated with anger through a sense of unfairness regarding male parental leave. In Japan, men in particular work long hours (5, 6) and there is a gender gap (6). Therefore, it is easy to think that child-rearing is the responsibility of women, and it is likely that colleagues in the workplace will have negative feelings toward men who take long-term parental leave. In addition, since the interpersonal fairness of supervisors is negatively associated with workplace stressors, it can be concluded that supervisors play an important role in workplaces where men take long periods of parental leave. Companies that promote male parental leave are actively working to reform work styles, such as by reducing working hours, and as a result, there are signs that supervisors are being encouraged to establish collaborative systems in workplaces where men take parental leave (8). To further facilitate the uptake of parental leave by men in Japan, it will be essential to thoroughly examine workplace factors such as colleagues’ stress levels and the role of supervisory support.

### 4.3 Limitations

This study did not directly address participants’ real-life experiences with actual male coworkers. In the future, if the number of men taking long-term parental leave increases, longitudinal studies targeting employees whose male coworkers have taken long-term parental leave will become possible. In addition, this study does not consider the age of the children, which is an attribute that may affect the responses. Parents of young, dependent children are likely to have vastly different needs and concerns compared to those whose children are no longer in their care. In the future, it will be necessary to distinguish between those who have children to care for and those whose children are already independent to conduct research.

## Data Availability

The raw data supporting the conclusions of this article will be made available by the authors, without undue reservation.
